# Oxidative Stress Markers in Cerebrospinal Fluid of Newly Diagnosed Multiple Sclerosis Patients and Their Link to Iron Deposition and Atrophy

**DOI:** 10.3390/diagnostics12061365

**Published:** 2022-06-01

**Authors:** Andrea Burgetova, Petr Dusek, Tomas Uher, Manuela Vaneckova, Martin Vejrazka, Romana Burgetova, Dana Horakova, Barbora Srpova, Jan Krasensky, Lukas Lambert

**Affiliations:** 1 Department of Radiology, First Faculty of Medicine, Charles University and General University Hospital in Prague, 120 00 Prague, Czech Republic; andrea.burgetova@vfn.cz (A.B.); petr.dusek@vfn.cz (P.D.); manuela.vaneckova@vfn.cz (M.V.); romana.burgetova@fnkv.cz (R.B.); jan.krasensky@vfn.cz (J.K.); 2 Department of Neurology, First Faculty of Medicine, Charles University and General University Hospital in Prague, 120 00 Prague, Czech Republic; tomas.uher@vfn.cz (T.U.); dana.horakova@vfn.cz (D.H.); barbora.srpova@vfn.cz (B.S.); 3 Institute of Medical Biochemistry and Laboratory Diagnostics, First Faculty of Medicine, Charles University and General University Hospital in Prague, 120 00 Prague, Czech Republic; martin.vejrazka@vfn.cz; 4 Department of Radiology, Third Faculty of Medicine, Charles University, 100 34 Prague, Czech Republic

**Keywords:** multiple sclerosis, magnetic resonance imaging, cerebrospinal fluid, oxidative stress, peroxiredoxin, neutrophil gelatinase-associated lipocalin

## Abstract

Oxidative stress has been implied in cellular injury even in the early phases of multiple sclerosis (MS). In this study, we quantified levels of biomarkers of oxidative stress and antioxidant capacity in cerebrospinal fluid (CSF) in newly diagnosed MS patients and their associations with brain atrophy and iron deposits in the brain tissue. Consecutive treatment-naive adult MS patients (*n* = 103) underwent brain MRI and CSF sampling. Healthy controls (HC, *n* = 99) had brain MRI. CSF controls (*n* = 45) consisted of patients with non-neuroinflammatory conditions. 3T MR included isotropic T1 weighted (MPRAGE) and gradient echo (GRE) images that were processed to quantitative susceptibility maps. The volume and magnetic susceptibility of deep gray matter (DGM) structures were calculated. The levels of 8-hydroxy-2′-deoxyguanosine (8-OHdG), 8-iso prostaglandin F2α (8-isoPG), neutrophil gelatinase-associated lipocalin (NGAL), peroxiredoxin-2 (PRDX2), and malondialdehyde and hydroxyalkenals (MDA + HAE) were measured in CSF. Compared to controls, MS patients had lower volumes of thalamus, pulvinar, and putamen, higher susceptibility in caudate nucleus and globus pallidus, and higher levels of 8-OHdG, PRDX2, and MDA + HAE. In MS patients, the level of NGAL correlated negatively with volume and susceptibility in the dentate nucleus. The level of 8-OHdG correlated negatively with susceptibility in the caudate, putamen, and the red nucleus. The level of PRDX2 correlated negatively with the volume of the thalamus and both with volume and susceptibility of the dentate nucleus. From MRI parameters with significant differences between MS and HC groups, only caudate susceptibility and thalamic volume were significantly associated with CSF parameters. Our study shows that increased oxidative stress in CSF detected in newly diagnosed MS patients suggests its role in the pathogenesis of MS.

## 1. Introduction

Multiple sclerosis (MS) is a chronic neuroinflammatory demyelinating disease with progressive deterioration of disability in the majority of untreated patients. Although the main pathological mechanism is an aberrant immunological response, there is a complex interaction of genetic and environmental factors as well as neuroprotective and repair mechanisms [1]. Oxidative stress and its modulation have been implied in the formation, sustenance, and termination of cell injury in MS [2,3].

The levels of oxidative stress markers can be estimated in patients from peripheral blood, cerebrospinal fluid (CSF), or locally by microdialysis. Since CSF is directly connected to extracellular space, its analysis may better reflect homeostasis in the brain microenvironment compared to peripheral blood analysis [4]. Oxidative stress markers including malondialdehyde (MDA), 4-hydroxyalkenals (HAE), isoprostane 8-iso-PGF_2α_ (8-isoPG) have been reported to be elevated in CSF of MS patients [1,5]. The 8-hydroxy-2-deoxyguanosine (8-OHdG), a product of oxidative DNA damage in CSF, has been implied in carcinogenesis, neurodegeneration, but not specifically in MS [6,7]. Peroxiredoxins represent a family of highly efficient peroxidases that are upregulated in astrocytes in MS lesions [4]. Neutrophil Gelatinase-Associated Lipocalin (NGAL) acts in multiple signaling pathways that regulate inflammation, iron homeostasis, and cell death [8]. The above-mentioned substances have been investigated in MS patients, but the evidence for their dysregulation is limited, especially in the early phases of MS.

Newly diagnosed MS patients already show accelerated tissue loss of deep grey matter (DGM), particularly in the thalamus [9]. Iron accumulation in DGM in MS patients is also an early phenomenon, which is already detectable at the transition from clinically isolated syndrome to clinically definite MS [10]. Iron concentration in the DGM correlates with clinical severity and predicts future disability [11,12]. Iron balance is tightly linked with inflammation and many proteins involved in cellular iron homeostasis are altered by inflammatory stimuli resulting in cellular iron retention [13]. Iron dysregulation is supposed to trigger oxidative stress. However, the link between oxidative stress, brain atrophy, and iron concentration in DGM structures in MS has not been sufficiently established.

The main aim of this study was to examine biomarkers of oxidative stress and antioxidant capacity in CSF in newly diagnosed untreated MS patients and their associations with the severity of brain atrophy and iron deposits in DGM.

## 2. Materials and Methods

This study (ClinicalTrials.gov ID: NCT03706118) was approved by the Ethics Committee of the General University Hospital in Prague (ID1018/17), it was carried out in accordance with the Declaration of Helsinki and all subjects signed informed consent.

### 2.1. Study Participants

MS patients (*n* = 103): de novo treatment-naive MS patients diagnosed between August 2017 and January 2020 underwent neurological examination including Expanded Disability Status Scale (EDSS), brain MRI, and CSF sampling. The inclusion criteria were: age ≥ 18 years and diagnosis of MS according to the 2017 McDonald criteria [14]. We excluded patients with major diseases affecting brain health and pregnant women.

Two different groups of controls were used for brain MRI and CSF biochemical comparisons. 

Healthy controls (HC) for brain MRI were recruited from the general community [15]. The controls were free of neurologic or other medical disorders affecting brain health and had a normal neurological examination. From the original group of 111 subjects, we selected 99 HC with an age range comparable to MS patients by excluding older subjects.

CSF controls: for CSF comparison, samples from 45 controls with different non-neuroinflammatory conditions were selected from our CSF biobank. The control group included 37 patients with non-inflammatory neurological disorders and 8 patients undergoing spinal anesthesia for urologic surgery.

### 2.2. Imaging Protocol

MRI examination was performed and processed as previously published [15]: Magnetization Prepared Rapid Gradient-Echo (MPRAGE, TE: 2.96 ms, TI 900 ms TR: 2300 ms, spatial resolution: 1 × 1 × 1 mm), gradient-echo (GRE, 6 TEs: 4.5–29.5 ms, evenly spaced, TR: 33 ms, spatial resolution: 0.94 × 0.94 × 0.94 mm), and Fluid-Attenuated Inversion Recovery (FLAIR, TE 397 ms, TI 1800 ms, TR 5000 ms, spatial resolution 1 × 1 × 1 mm) pulse sequences were acquired on a 3T MRI scanner (Siemens Skyra 3T, Siemens Healthcare, Erlangen, Germany) with a 20 channel head coil. 

### 2.3. Image Processing

**Figure 1:**
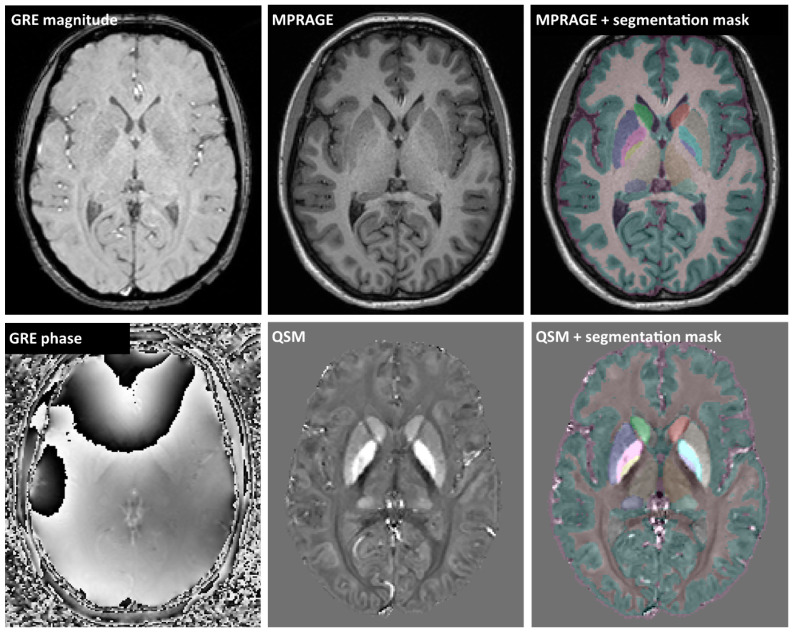
Example MRI images: gradient-echo (GRE) magnitude and phase images were processed to quantitative susceptibility map (QSM). Multi-atlas segmentation employed coregistered QSM and MPRAGE images to generate masks of deep grey matter structures, cerebral white and grey matter, and CSF.

GRE images were processed to quantitative susceptibility mapping (QSM) maps, co-registered with MPRAGE using SPM12 running under Matlab v. 2020a (The Math Works, Inc., Natick, MA, USA). Co-registered skull-stripped QSM and MPRAGE images entered a fully automated multi-atlas segmentation pipeline using dual (QSM and T1-weighted) contrast for delineation of DGM nuclei implemented with a cloud-based platform (www.mricloud.org) (Figure 1) [16]. Segmentation of DGM structures in all subjects was visually checked for errors, particularly for potential misclassifications of demyelinating lesions in the DGM. Volumes were adjusted to the total intracranial volume (TIV) and reported as the sum of bilateral structures. QSM measurements were referenced to the susceptibility of a manually drawn region in the parietooccipital white matter avoiding white-matter lesions [15]. White matter lesions were segmented by the lesion growth algorithm [17] as implemented in the LST toolbox version 3.0.0 (www.statistical-modelling.de/lst.html) for SPM. The optimal initial threshold (κ) was determined by visual inspection and set to 0.15. Lesion segmentation in all subjects were visually checked and false-positive voxels were manually deleted.

### 2.4. CSF Assays

CSF was drawn from L5-S1, L4-5, or L3-4 interspace in an upright sitting position in the morning hours using a standard sterile preparation and 20 G atraumatic needle. A total of 20–25 mL of CSF and 5 mL volume of blood were obtained. CSF was immediately centrifuged at 3000× *g* rpm and 4 °C; the supernatant was aliquoted and frozen at −80 °C.

For CSF assays, the samples were centrifuged after thawing and kept on ice. All samples were assayed in duplicates.

8-iso prostaglandin F_2α_ (8-isoPG, 8-isoprostane) was determined immunochemically by a non-competitive ELISA with biotinylated anti-human 8-isoPG antibody. Human 8-iso prostaglandin ELISA kit (cat. No. MBS160287, MyBioSource, Inc., San Diego, CA, USA) was employed. The calibration ranged from 0 to 640 ng/L and was linear throughout the whole range.

Neutrophil gelatinase-associated lipocalin (NGAL, lipocalin-2) was assayed using a non-competitive ELISA (cat. No. MBS5644140, MyBioSource, Inc., San Diego, CA, USA). Samples were diluted 10 times prior to use. The calibration ranged from 0 to 10 ng/mL and was linear in the whole range.

Peroxiredoxin-2 (PRDX2) was determined immunochemically by a non-competitive ELISA with biotinylated anti-human PRDX2 antibody (cat. No. ELH-PRDX2, RayBiotech, Norcross, GA, USA). Samples were diluted 5 times prior to use. The calibration ranged from 0 to 12.8 ng/mL. Polynomic regression of 2nd order was employed to estimate the concentrations of samples.

Products of lipid peroxidation were assayed by a colorimetric assay based on N-methyl-2-phenylindole (Bioxytech LPO-586 kit, cat. No. 21012, OXIS International, Inc., Foster City, CA, USA). Methanesulphonic acid was used as the acid solvent in the procedure for simultaneous determination of malondialdehyde and 4-hydroxyalkenals (MDA + HAE). Samples for MDA + HAE assay were stabilized with butylated hydroxytoluene (BHT; 20 µL of 0.2% BHT was added to 200 µL of CSF immediately after sampling, before freezing to −80 °C). In the assay, tetramethoxypropane was used as the calibrator in concentrations from 0 to 4 µmol/L.

8-hydroxy-2′-deoxyguanosine (8-OHdG) was assayed by a competitive ELISA (Bioxytech 8-OHdG EIA Kit, cat. No. 21026, OXIS International, Inc., Foster City, CA, USA). The calibration was performed for concentrations 0.5 to 8 ng/mL and was linear in this range.

In MS patients, CSF samples in sufficient quantity for the analysis of 8-OHdG, 8-isoPG, NGAL, and PRDX2 were available in 62 patients and for the analysis of MDA + HAE in 37 patients.

### 2.5. Statistical Analysis

Statistical analysis was performed in SPSS (IBM, Armonk, NY, USA). Dichotomous and continuous variables were compared using the Fisher test, the *t*-test, the Mann-Whitney test, or the generalized linear model (GLM) as appropriate according to the distribution of the data. Grubb’s test was used to identify significant outliers (*p* < 0.05). Age and sex-adjusted partial correlation coefficients among biochemical markers and deep gray matter volumes and susceptibilities in newly diagnosed MS patients were calculated. *p* values were adjusted to age and sex. A *p*-value below 0.05 was considered significant.

## 3. Results

### 3.1. Comparison of MS Patients and HC MRI

Brain volumetry and quantification of susceptibility in the DGM structures are shown in Table 1. Compared to HC, MS patients had lower brain parenchymal fraction (*p* = 0.001), a smaller volume of thalamus (*p* < 0.001), pulvinar (*p* < 0.001) and putamen (*p* = 0.002), and higher susceptibility in the caudate nucleus (*p* = 0.041) and globus pallidus internus (*p* = 0.015).

### 3.2. Comparison of CSF Biochemical Markers in MS Patients and Controls

CSF was available in 62 newly diagnosed MS patients. The control CSF group was older compared to MS patients (33 ± 9 vs. 40 ± 12 years, *p* < 0.001). Patients with MS had higher values of 8-OHdG (*p* = 0.041), PRDX2 (*p* = 0.015), and lipoperoxidation markers (MDA + HAE; *p* = 0.003) compared to HC (Table 2).

### 3.3. Correlations between CSF Biochemical Markers and MRI Parameters

**Figure 2:**
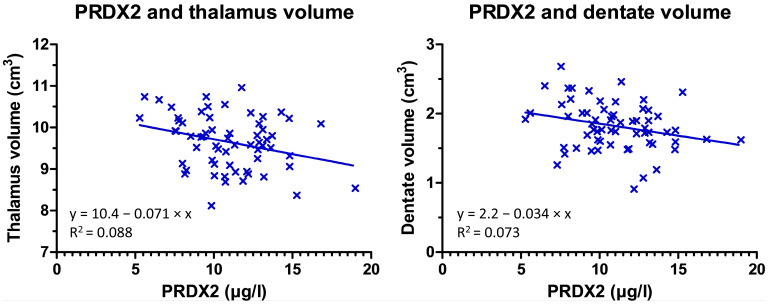
Scatter plot and regression line show negative correlation between PRDX2 and volume of the thalamus (**left**) and the dentate nucleus (**right**).

In MS patients, the correlations between CSF and MRI parameters were low to moderate. From MRI parameters with significant differences between MS and HC groups, only caudate susceptibility and thalamic volume were significantly associated with CSF parameters. The level of 8-OHdG correlated negatively with the susceptibility in the caudate, putamen, and the red nucleus. The level of 8-isoPG correlated negatively with susceptibility in the globus pallidus externus. The level of NGAL correlated negatively with susceptibility and volume in the dentate. The level of PRDX2 correlated negatively with the volume of the thalamus and both susceptibility and volume of the dentate nucleus (Figure 2). There was a negative correlation between lipoperoxidation markers and the volume of the globus pallidus and subthalamic nucleus (Table 3). None of the biochemical markers was significantly associated with T2 lesion volume, lesion count, or EDSS.

## 4. Discussion

In this study, we found that newly diagnosed MS patients show higher susceptibility in caudate nucleus and globus pallidus internus than healthy controls which is consistent with excessive iron accumulation in MS patients. These patients have higher levels of oxidative stress markers in CSF, which is correlated with susceptibility and volume of deep gray matter structures.

Oxidative stress has been implied in the pathogenesis of autoimmune inflammatory diseases such as MS [4,5,18]. Increased release of reactive oxygen species has been shown to cause extensive myelin damage in early MS [19]. Endogenous cytoprotective enzymes such as peroxiredoxin, glutathione reductase, or catalase counteract oxidative damage, regulate redox signaling pathways, and modulate inflammatory and immune responses [20]. The balance between reactive oxygen species production and antioxidant defense is crucial in preventing structural damage to the central nervous system [21].

**Peroxiredoxins** are a family of highly efficient peroxidases that catalyze the reduction of hydrogen peroxide, peroxynitrite, and alkyl hydroperoxides [20]. For example, PRDX2 is upregulated in astrocytes of MS lesions and its expression correlates with the degree of inflammation and oxidative stress [4]. There are four subtypes of peroxiredoxins that are expressed in the human brain [22]. PRDX2 is mainly expressed in astrocytes. Its expression is more abundant in MS lesions (especially in their rim), where activation of microglia and CD3 T cells is also present [4]. This is consistent with the finding of increased CSF levels of PRDX2 observed in MS patients in our study. PRDX2 expression level is positively correlated with the degree of inflammation and oxidative stress. Although PRDX2 protects neurons from oxidative stress and inflammatory damage, its increase in CSF may represent a negative prognostic marker that reflects ongoing oxidative stress. The negative correlation of PRDX2 with the volume of the thalamus may thus indicate the contribution of oxidative stress to thalamic atrophy in MS, which is also a strong predictor of disability progression [23].

The **8-hydroxy-2-deoxyguanosine** (8-OHdG) is the most abundant product of oxidative damage to DNA. Its role as an indicator of oxidative stress has been suggested in many conditions including neurodegenerative diseases [7]. Its elevated levels in CSF are found in patients with Parkinson’s disease, especially in its early stage [24]. Khajenobar et al. compared the levels of 8-OHdG in CSF of MS patients and healthy controls but did not find any significant difference [6]. Counterintuitively, they reported decreased levels of 8-OHdG in the serum of MS patients. On the contrary, Tasset et al. found higher 8-OHdG serum concentrations and their association with the severity of the MS [25]. We also found higher concentrations of 8-OHdG in CSF of newly diagnosed MS patients supporting the association between DNA oxidative stress and MS.

The **isoprostane 8-iso-PGF2α** is a biomarker of lipid peroxidation [5,26]. Mir et al. reported increased CSF levels of 8-isoPG in a large cohort of MS patients, with even higher levels in those with secondary progressive form than in relapsing-remitting MS (RRMS) [5]. Increased CSF 8-isoPG levels correlate with disease severity as assessed by EDSS [27]. We believe that a lack of association between CSF 8-isoPG as well as other CSF markers and EDSS in our study is likely caused by the early phase and very mild severity of our MS group.

**Neutrophil Gelatinase-Associated Lipocalin** (NGAL, also known as Lipocalin-2, or LCN2) is involved in several inflammatory pathways [28]. Its increased concentration in CSF is reported in patients with MS, especially those with secondary progressive MS and primary progressive MS compared to RRMS [8]. NGAL levels decrease after natalizumab treatment. Khalil et al. report decreased levels of NGAL in CSF of MS patients and their positive association with iron accumulation in the basal ganglia measured using R2* relaxometry [29]. In our study, the difference between MS patients and healthy controls was not significant. However, we showed a negative correlation of NGAL levels with the susceptibility in the dentate nucleus. These disparate findings may be caused by different methods of iron estimation. Increased susceptibility in MS, as compared to R2* relaxometry, reflects not only iron accumulation but also demyelination. This factor may bias the analyses of associations between NGAL and other examined CSF parameters with DGM iron. Additionally, the wide spectrum of NGAL actions may contribute to its different pathogenetic effects depending on disease stage. These effects include induction of neuronal cell death and modulation of iron concentration and its storage in cells, which is one of the features of neurodegeneration in MS, as previously shown by our study group [30,31,32] and others [10].

Both **malondialdehyde and hydroxyalkenals** are markers of lipid peroxidation [33]. The brain tissue is highly sensitive to reactive oxygen species due to the increased oxygen consumption and a high concentration of polyunsaturated fatty acids which are sensitive to lipid peroxidation [34]. The process of iron-dependent accumulation of lipid peroxidation is referred to as ferroptosis and leads to the accumulation of toxic reactive oxygen species. We found higher CSF levels of MDA + HAE in MS patients compared to healthy controls and an association with lower volumes of GPI, GPE, and the subthalamic nucleus. Yet, we did not find significant atrophy of these structures in our MS cohort. This finding may be thus consistent with prodromal neurodegeneration of globus pallidus and subthalamic nucleus in a subgroup of MS patients with increased lipid peroxidation. On the other hand, no association between MDA-HAE and iron levels was observed arguing against the theory that paramagnetic iron measured by QSM is directly involved in ferroptosis.

Consistently with previous studies, our MR data show that newly diagnosed MS patients already have atrophy of the thalamus, thalamic pulvinar, and putamen [35]. Caudate and globus pallidus show significantly higher susceptibility in MS patients consistent with iron accumulation [31]. Additionally, we show that the increased susceptibility in globus pallidus in newly diagnosed MS patients is driven by changes in the internal pallidum.

Our study has some limitations. The increase of CSF levels of 8-OHdG, PRDX2, and MDA + HAE may also be driven by the disruption of blood-brain barrier and not only by their increased CSF content. Furthermore, circadian fluctuations of CSF levels of the investigated biomarkers are known. On the other hand, lumbar punctures were performed at similar times of the day limiting the effect of circadian fluctuations. Although magnetic susceptibility is a marker of iron concentration, it is also affected by other substances such as myelin. Finally, although the age difference between MS patients and CSF controls was significant, we did not find any age dependence of CSF parameters and adjusted statistical analyses for age whenever appropriate.

## 5. Conclusions

Early MS patients show atrophy of the thalamus, thalamic pulvinar, and putamen and iron accumulation in the caudate and GPI. Increased levels of PRDX2, MDA + HAE, and 8-OHdG in CSF in newly diagnosed MS patients may suggest the role of oxidative stress in the pathogenesis of MS. The association between PRDX2 levels and the decreasing volume of the thalamus as well as between 8-OHdG and increasing susceptibility in the caudate nucleus underlines the connection between neuroinflammation, oxidative stress, and tissue loss in MS. PRDX2 and 8-OHdG may be considered as biomarkers in longitudinal MS studies. Further investigation of oxidative stress biomarkers in MS is needed due to their potential role in predicting and monitoring disease progression and response to therapy.

## Figures and Tables

**Table 1:** Comparison of DGM volumes and susceptibilities in MS patients and controls.

	MS (*n* = 103)		MRI Controls (*n* = 99)		
	Mean	Std. Deviation	Mean	Std. Deviation	*p*-Value
Sex (male/female)	28/75		38/61		0.090
Age (years)	32.5	8.0	33.7	8.3	0.305
EDSS (median, IQR)	2	1.5–2.5	-	-	-
Lesion load (cm^3^)	2.7	5.3	-	-	-
Lesion count (median, IQR)	7	4–20	-	-	-
Brain parenchymal fraction (%)	80.3	3.4	81.5	2.8	**0.001**
**DGM Volumes (cm^3^)**					
caudate	8.0	0.7	8.1	0.6	0.240
GPI	1.1	0.1	1.1	0.1	0.230
GPE	3.1	0.3	3.2	0.3	0.528
putamen	8.7	0.7	9.0	0.8	**0.002**
thalamus	9.6	0.6	9.9	0.6	**<0.001**
pulvinar	2.3	0.3	2.5	0.3	**<0.001**
subthalamic nucleus	0.3	0.1	0.4	0.1	0.310
substantia nigra	1.3	0.1	1.4	0.2	0.163
red nucleus	0.6	0.1	0.6	0.1	0.710
dentate	1.8	0.4	1.8	0.3	0.711
**DGM Susceptibilities (ppb)**					
Caudate	24.5	5.8	23.0	4.7	**0.041**
GPI	53.1	6.4	50.9	5.7	**0.015**
GPE	62.8	7.7	62.2	6.8	0.594
Putamen	23.6	6.7	23.6	6.1	0.977
Thalamus	0.9	2.3	1.0	2.0	0.728
Pulvinar	17.9	5.2	18.3	5.2	0.582
subthalamic nucleus	41.9	7.3	43.2	6.9	0.214
substantia nigra	53.4	7.3	52.2	6.6	0.099
red nucleus	39.2	7.8	39.7	8.1	0.639
Dentate	37.4	8.6	36.9	9.8	0.693

DGM, deep gray matter; IQR, interquartile range; EDSS, expanded disability status scale; GPI, globus pallidus internus; GPE, globus pallidus externus; ppb, parts per billion. *p*-values are adjusted for age and sex. Significant differences are in bold.

**Table 2:** Comparison of CSF biochemical markers in MS patients and controls.

	MS (*n* = 62)		CSF Controls (*n* = 45)		
	Mean	Std. Deviation	Mean	Std. Deviation	*p*-Value
Sex (male/female)	19/43		20/25		0.143
Age (years)	33.3	8.6	40.2	11.6	<0.001
CSF sampling to MRI interval (months)	1.1	3.9	n.d	n.d.	n.d.
Cerebrospinal Fluid Analysis					
8-OHdG (ng/mL)	0.112 (median = 0)	0.310 (IQR = 0 to 0)	0.026 (median = 0)	0.122 (IQR = 0 to 0)	**0.041** ^1^
8-isoPG (ng/L)	44.319	13.611	41.071	9.679	0.447
NGAL (ng/mL)	4.366	2.085	4.968	2.226	0.473
PRDX2 (ng/mL)	10.966	2.961	9.437	3.945	**0.015**
MDA + HAE (µmol/L)	0.605 ^2^	0.264 ^2^	0.448	0.153	**0.003**

^1^ Mann-Whitney U test; other analyses performed using GLM with age and sex as covariates; ^2^ *n* = 37; CSF, cerebrospinal fluid; 8-OHdG, 8-hydroxy-2′-deoxyguanosine; 8-isoPG, 8-isoprostane; NGAL, neutrophil gelatinase-associated lipocalin; PRDX2, peroxiredoxin-2; MDA + HAE, malondialdehyde and hydroxyalkenals; n.d., not determined. *p*-values are adjusted for age and sex. Significant differences are in bold.

**Table 3:** Age and sex-adjusted partial correlation coefficients among biochemical markers and deep gray matter volumes and susceptibilities in early MS patients.

Structure	8-OHdG		8-isoPG		NGAL		PRDX2		MDA + HAE	
	*r*	*p*	*r*	*p*	*r*	*p*	*r*	*p*	*r*	*p*
**Volume**										
caudate	0.017	*0.899*	−0.147	*0.271*	−0.170	*0.195*	−0.236	*0.069*	−0.328	*0.055*
globus pallidus int.	0.175	*0.181*	−0.020	*0.879*	0.039	*0.765*	−0.005	*0.971*	**−0.459**	***0.006***
globus pallidus ext.	0.195	*0.136*	−0.199	*0.134*	−0.081	*0.539*	−0.083	*0.529*	**−0.433**	***0.009***
putamen	0.115	*0.380*	0.022	*0.869*	−0.137	*0.297*	−0.069	*0.602*	−0.008	*0.963*
thalamus	0.074	*0.575*	−0.028	*0.833*	−0.045	*0.733*	**−0.341**	***0.008***	0.025	*0.886*
pulvinar thalami	0.129	*0.328*	0.036	*0.786*	−0.067	*0.613*	−0.240	*0.064*	0.020	*0.909*
subthalamic nucleus	0.032	*0.806*	−0.189	*0.155*	0.008	*0.950*	0.011	*0.934*	**−0.374**	***0.027***
substantia nigra	0.002	*0.990*	−0.115	*0.391*	−0.145	*0.268*	−0.112	*0.393*	−0.229	*0.187*
red nucleus	0.046	*0.724*	−0.152	*0.256*	−0.036	*0.786*	−0.034	*0.795*	−0.299	*0.081*
dentate	−0.014	*0.916*	−0.097	*0.471*	**−0.292**	***0.023***	**−0.291**	***0.024***	−0.261	*0.130*
**Susceptibility**										
caudate	**−0.283**	***0.026***	−0.098	*0.450*	0.009	*0.942*	−0.042	*0.747*	0.200	*0.234*
globus pallidus int	0.029	*0.821*	−0.141	*0.276*	0.120	*0.352*	0.124	*0.339*	0.207	*0.220*
globus pallidus ext	−0.037	*0.777*	**−0.276**	***0.030***	0.017	*0.894*	0.097	*0.452*	0.283	*0.089*
putamen	**−0.396**	***0.001***	−0.095	*0.462*	0.031	*0.808*	−0.058	*0.654*	0.213	*0.205*
thalamus	0.139	*0.280*	0.103	*0.426*	−0.055	*0.669*	−0.099	*0.446*	−0.088	*0.603*
pulvinar thalami	−0.172	*0.181*	−0.016	*0.900*	−0.129	*0.317*	−0.245	*0.055*	0.024	*0.888*
subthalamic nucleus	−0.141	*0.275*	−0.215	*0.093*	0.016	*0.899*	0.092	*0.476*	0.285	*0.088*
substantia nigra	−0.101	*0.433*	−0.107	*0.407*	0.052	*0.686*	0.190	*0.139*	0.296	*0.076*
red nucleus	**−0.309**	***0.015***	−0.075	*0.561*	0.016	*0.904*	−0.010	*0.941*	0.295	*0.077*
dentate	−0.067	*0.604*	−0.154	*0.231*	**−0.314**	***0.013***	**−0.293**	***0.021***	0.011	*0.947*

8-OHdG, 8-hydroxy-2′-deoxyguanosine; 8-isoPG, 8-isoprostane; NGAL, Neutrophil gelatinase-associated lipocalin; PRDX2, peroxiredoxin-2; MDA + HAE, malondialdehyde and hydroxyalkenals; r, Spearman correlation coefficient; p, *p*-value (*p*-values < 0.05 in bold); volume adjusted to total intracranial volume.

## Data Availability

Data are available upon a reasonable request to the study investigators.
